# An interpretable machine learning model for stroke recurrence in patients with symptomatic intracranial atherosclerotic arterial stenosis

**DOI:** 10.3389/fnins.2023.1323270

**Published:** 2024-01-08

**Authors:** Yu Gao, Zi-ang Li, Xiao-yang Zhai, Lin Han, Ping Zhang, Si-jia Cheng, Jun-yan Yue, Hong-kai Cui

**Affiliations:** ^1^Department of Radiology Center, The First Affiliated Hospital of Xinxiang Medical University, Xin Xiang, China; ^2^Department of Neurology, The First Affiliated Hospital of Xinxiang Medical University, Xin Xiang, China; ^3^Department of Neurointerventional Center, The First Affiliated Hospital of Xinxiang Medical University, Xin Xiang, China

**Keywords:** stroke recurrence, HR-VWI, machine learning, SICAS, plaque

## Abstract

**Background and objective:**

Symptomatic intracranial atherosclerotic stenosis (SICAS) is the most common etiology of ischemic stroke and one of the main causes of high stroke recurrence. The recurrence of stroke is closely related to the prognosis of ischemic stroke. This study aims to develop a machine learning model based on high-resolution vessel wall imaging (HR-VWI) to predict the risk of stroke recurrence in SICAS.

**Methods:**

This study retrospectively collected data from 180 SICAS stroke patients treated at the hospital between 2020.01 and 2022.01. Relevant imaging and clinical data were collected, and follow-up was conducted. The dataset was divided into a training set and a validation set in a ratio of 7:3. We employed the least absolute shrinkage and selection operator (LASSO) regression to perform a selection on the baseline data, laboratory tests, and neuroimaging data generated by HR-VWI scans collected from the training set. Finally, five machine learning techniques, including logistic regression model (LR), support vector machine (SVM), Gaussian naive Bayes (GNB), Complement naive Bayes (CNB), and k-nearest neighbors algorithm (kNN), were employed to develop a predictive model for stroke recurrence. Shapley Additive Explanation (SHAP) was used to provide visualization and interpretation for each patient. The model’s effectiveness was evaluated using average accuracy, sensitivity, specificity, precision, f1 score, PR curve, calibration curve, and decision curve analysis.

**Results:**

LASSO analysis revealed that “history of hypertension,” “homocysteine level,” “NWI value,” “stenosis rate,” “intracranial hemorrhage,” “positive remodeling,” and “enhancement grade” were independent risk factors for stroke recurrence in SICAS patients. In 10-fold cross-validation, the area under the curve (AUC) ranged from 0.813 to 0.912 in ROC curve analysis. The area under the precision-recall curve (AUPRC) ranged from 0.655 to 0.833, with the Gaussian Naive Bayes (GNB) model exhibiting the best ability to predict stroke recurrence in SICAS. SHAP analysis provided interpretability for the machine learning model and revealed essential factors related to the risk of stroke recurrence in SICAS.

**Conclusion:**

A precise machine learning-based prediction model for stroke recurrence in SICAS has been established to assist clinical practitioners in making clinical decisions and implementing personalized treatment measures.

## Introduction

Acute ischemic stroke is a common cerebrovascular disease with high incidence, disability, and mortality rates (Zhu et al., 2016). Acute ischemic stroke is a common cerebrovascular disease with a high incidence, disability, and mortality rate. Stroke is the second leading cause of death worldwide and the primary cause of death in Chinese populations in recent years. In Western countries, the proportion of ischemic stroke caused by intracranial atherosclerotic narrowing is about 10–15%, while in Asia, this proportion reaches up to 46.6% (Zheng et al., 2022). However, patients with intracranial atherosclerotic narrowing have a high risk of stroke recurrence. With the progress of medical technology, people have gained some understanding of the risk factors for stroke recurrence. Understanding the risk and developing individualized treatment for stroke patients will be the key to future medical research.

In recent years, HR-VWI has played a crucial role in the precise prevention and treatment of acute ischemic stroke. Various imaging techniques, such as CTA, MRA, and DSA, are commonly used for assessing cerebral vessels in stroke patients. While these techniques effectively depict intraluminal blood flow, they cannot comprehensively evaluate plaque location, characteristics, and the degree of luminal stenosis in atherosclerotic stenosis-related strokes. In contrast, HR-VWI addresses these limitations in conventional imaging and has become a common adjunctive examination for symptomatic intracranial atherosclerotic stenosis (SICAS) (Vranic et al., 2021; Tang et al., 2022). Previous studies have demonstrated a significant association between plaque information derived from HR-VWI, such as intra-plaque hemorrhage, plaque enhancement grade, positive remodeling, and the normalized wall index (NWI), with stroke recurrence (Roquer et al., 2011; Ran et al., 2020). Moreover, HR-VWI exhibits superior sensitivity and specificity compared to other risk factors.

Since the twenty-first century, artificial intelligence (AI) has undergone continuous development, and machine learning, as an AI methodology, has increasingly been integrated into medical research. Machine learning has successfully been employed in diagnosing and predicting various diseases by extracting relevant information and uncovering hidden correlations among parameters from vast datasets (Singal et al., 2013; Wu et al., 2020). Therefore, this study aims to develop and validate a machine-learning model that analyzes and predicts the risk of stroke recurrence in SICAS.

## Materials and methods

### Population

This study included 180 SICAS stroke patients randomly collected from the hospital database between 2020.01 and 2022.01. The relevant imaging and clinical data were extracted. The inclusion criteria were as follows: (1) intracranial atherosclerotic stenosis ranging from 30 to 99%; (2) presence of symptoms of ischemic stroke or transient ischemic attack (TIA); (3) acute infarction located in the same-side area of intracranial atherosclerotic stenosis as demonstrated by diffusion-weighted imaging (DWI); (4) all patients underwent HR-VWI examination.

Exclusion criteria are: (1) narrowing of the carotid artery >50% on ipsilateral ultrasound or magnetic resonance angiography (MRA) or computed tomography angiography (CTA); (2) non-atherosclerotic vascular diseases such as dissection, vasculitis, or stroke; (3) evidence of cardiac embolism: intracardiac thrombus detected by transesophageal echocardiography; (4) poor imaging quality that affects plaque evaluation or the presence of contraindications to MRI; and (5) patients undergoing percutaneous transluminal angioplasty and stent placement.

Baseline data information includes gender, age, BMI, smoking history, alcohol consumption history, hypertension, diabetes, myocardial infarction, atrial fibrillation, previous history of cerebrovascular disease, and NIHSS score at admission. Laboratory examination information includes total cholesterol, triglycerides, LDL, HDL, apolipoprotein A, apolipoprotein B, fibrinogen, blood glucose, and homocysteine. Follow-up: Using the forms of phone or face-to-face consultations, the median follow-up time was 18 months (12–30 months), and the patient’s condition was registered. The determination of stroke recurrence requires the evaluation of two or more senior clinicians (≥10 years of experience), and the diagnostic criteria are as follows (Coull and Rothwell, 2004): (1) A sudden onset of new focal neurological dysfunction lasting for more than 24 h. (2) Focal neurological dysfunction that lasts for less than 24 h but is confirmed by imaging as acute cerebral infarction, and there is no bleeding lesion on head CT or MRI. (3) Sudden deterioration of neurological function with a increase in the NIHSS (National Institute of Health Stroke Scale) score of 4 points. (4) exclusion of cerebral hemorrhage, tumors, and other causes.

### MRI protocol

All patients underwent examinations using a 16-channel head and neck combined coils together with Philips 3.0 T magnetic resonance imaging (MRI) equipment. Each patient first received routine magnetic resonance (MR) head and skull scans, including diffusion-weighted imaging (DWI), three-dimensional time-of-flight MR angiography (3D-TOF MRA), T1-weighted imaging (T1WI), and T2-weighted imaging (T2WI). Subsequently, a 3D high-resolution MRI (HR-MRI) scan was performed. Initially, a three-dimensional volume T1-weighted isotropic turbo spin echo acquisition (3D T1W-VISTA) was conducted based on the MRA images. Following the completion of the imaging, gadobutrol (Gadavist, Bayer, 0.1 mmol/kg) was injected, followed by a second 3D T1W-VISTA scan. The scanning parameters for each sequence were as follows: (1) DWI sequence: TR 2,194 ms, TE 86 ms, FOV 230 mm × 230 mm × 109 mm, slice thickness 5 mm, voxel size 1.5 mm × 1.89 mm × 5 mm, matrix size 152 × 122 × 17, interslice spacing 1.5 mm, and scan time 33 s; (2) 3D-TOF MRA sequence: TR 19 ms, TE 3.5 ms, FOV 200 mm × 158 mm × 89 mm, slice thickness 1.2 mm, voxel size 0.65 mm × 0.94 mm × 1.2 mm, matrix size 308 × 168 × 148, interslice spacing −0.6 mm, and scan time 2 min 52 s; (3) T1WI sequence: TR 2373 ms, TE 20 ms, FOV 230 mm × 189 mm × 109 mm, slice thickness 5 mm, voxel size 0.8 mm × 1.05 mm × 5 mm, matrix size 288 × 178 × 17, interslice spacing 1.5 mm, and scan time 1 min 30 s; (4) T2WI sequence: TR 2756 ms, TE 105 ms, FOV 230 mm × 230 mm × 109 mm, slice thickness 5 mm, voxel size 0.95 mm × 0.95 mm × 5 mm, matrix size 244 × 244 × 17, interslice spacing 1.5 mm, and scan time 44 s; (5) 3D T1W-VISTA sequence: TR 600 ms, TE 31 ms, FOV 250 mm × 161 mm × 60 mm, slice thickness 0.6 mm, voxel size 0.8 mm × 0.8 mm × 0.8 mm, matrix size 312 × 201 × 150, interslice spacing −0.4 mm, and scan time 3 min 36 s.

### Image plaque analysis

The culprit plaque is defined as the lesion on the same side of the fresh stroke in the DWI image. The narrowest lesion is selected for analysis if multiple plaques exist in the same vascular distribution area. The acquired 3D HR-VWI images were reconstructed vertically along the long axis of the vessel where the culprit lesion was located, following the guidelines of the American Society of Neuroradiology Vessel Wall Imaging (Saba et al., 2018). This reconstruction eliminates deviations between different devices and better displays the plaque and vessel wall status in the coronal, sagittal, and axial planes. Two experienced neuroradiologists (Yue and Zhai) performed the analysis without access to the patient’s clinical data. The analysis primarily focuses on plaque identification and enhancement. The software TS-Vessel·Explore (TSimaging; Healthcare China, Beijing) is used for post-processing and data analysis. The HR-VWI images are imported into the post-processing software and reconstructed along a cross-section perpendicular to the long axis of the vessel, with a magnification of 400%. A manual tracing model is employed to outline the vessel wall’s outer contour and the lumen’s inner contour, with the software automatically measuring the corresponding vessel area (VA) and lumen area (LA). The plaque with the narrowest stenosis at the lumen was chosen as the culprit plaque for measurement.

The plaque responsible for the narrowest part of the lumen is selected for measurement. For the reference level, the VA and LA are prioritized from the corresponding lumen section without obvious plaques near it, followed by the related lumen section far away.

The degree of vascular stenosis is calculated using the following formulas: stenosis rate = (1 − LAmin/LAreference) × 100%; wall area (WA) = VA − LA; plaque area (PA) = WAmin − WAreference; remodeling index (RI) = VAmin/VAreference, with RI ≥ 1.05 indicating positive remodeling and RI ≤ 0.95 indicating negative remodeling (Teng et al., 2016).

The normalized wall index (NWI) is calculated as WA/VA. In the enhanced T1W VISTA image, the signal intensity of the pituitary gland is used as the reference, with no change in plaque signal considered grade 0; enhancement lower than the pituitary gland is grade 1; and similar enhancement to the normal pituitary gland is grade 2 (Qiao et al., 2014). Intraplaque hemorrhage (IPH) is defined as T1WI signal intensity higher than 150% of the adjacent muscle tissue signal.

### Statistical analysis

Software including R (version 3.6.8) and Python (version 3.7), A result with *p* < 0.05 indicates statistical significance.

### Development, evaluation, and interpretation of machine learning models

In the collected dataset, 130 cases (72.2%) of patients did not experience a recurrent stroke, while 50 cases (37.8%) did. The dataset was divided into training and validation sets in a ratio of 7:3. The LASSO regression algorithm, which involves shrinking and selecting features (non-zero coefficients), was employed to choose the most effective features from data collected from the training set. The regularization parameter lambda was adjusted to control the strength of regularization, and 10-fold cross-validation was used to select features. This study utilized five machine learning algorithms, namely logistic regression (LR), support vector machine (SVM), Gaussian naive Bayes (GaussianNB), Complement naive Bayes (ComplementNB), and k-nearest neighbors algorithm (kNN), to predict recurrent strokes in SICAS. For the training set, k-fold cross-validation (*k* = 10) was employed as a resampling technique, and grid search was used to fine-tune hyperparameters. The training set was utilized for parameter adjustment, and the validation set was used to evaluate the system’s performance. The clinical value of the predictive model was assessed through three measures: discriminative ability, calibration, and clinical effectiveness. Firstly, a quantitative analysis of model discriminative ability was conducted using ROC and PR curves. Subsequently, the calibration of the model and the extent of prediction bias toward actual events were assessed using calibration curves. Furthermore, clinical net benefit was evaluated through decision curve analysis (DCA). In addition, the study assessed the accuracy, sensitivity, specificity, F1 score and kappa using confusion matrix indicators for the five models.

**Figure 1:**
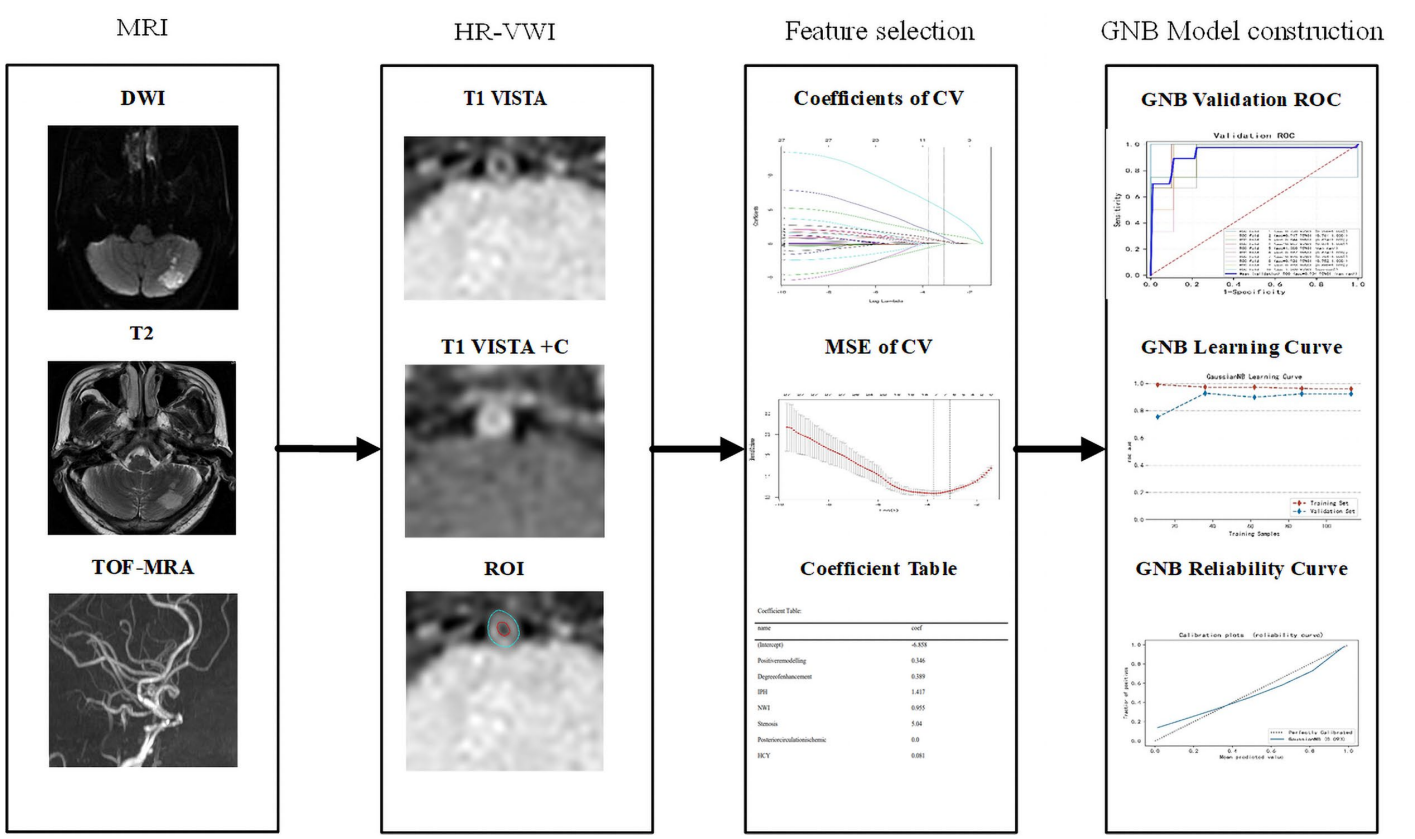
Workflow of machine learning model construction. CV, cross validation; MSE, mean square error; GNB, Gaussian naive Bayes; DCA, decision curve analysis.

The model explanation primarily relies on the use of the SHAP method. Initially applied in cooperative game theory to address the problem of allocation equilibrium, this method offers the possibility of replacing machine learning models with limited interpretability by incorporating concepts such as Shap values for explanatory purposes. The Shap value, an allocation algorithm, ensures a fair assignment of the outcome (prediction result) to various features, compensating for the lack of interpretability in machine learning. In explaining machine learning models, the Shap value indicates the individual input feature’s significance in contributing to the model’s predicted value. A higher Shap value reflects a more significant influence of the input feature on the prediction result. The flowchart for building and validating a machine learning model is shown in Figure 1.

## Results

### Patient characteristics

This study enrolled a total of 180 patients who met the inclusion criteria. The dataset included 130 cases (72.2%) of patients without recurrent stroke and 50 cases (37.8%) of patients with recurrent stroke. The average age of the participants was 60 years. There were 122 male (67.8%) participants and 58 female participants (32.2%). The study observed that the HYC levels in the laboratory tests were higher in the recurrent stroke group compared to the non-recurrent stroke group (20.2 vs. 13.5, *p* < 0.001). Additionally, in the imaging data, the recurrent stroke group exhibited higher NWI values (0.9 vs. 0.7, *p* < 0.001), a greater degree of vascular stenosis (70% vs. 40%, *p* < 0.001), increased plaque enhancement levels (Grade 0: 10.0% vs. 49.2%; Grade 1: 44.0% vs. 36.2%; Grade 2: 46.0% vs. 14.6%, *p* < 0.001), a higher incidence of intraplaque hemorrhage (62.0% vs. 11.5%, *p* < 0.001), and a higher proportion of positive remodeling (46.0% vs. 28.5%, *p* < 0.001) compared to the non-recurrent stroke group (Table 1).

**Table 1:** Baseline characteristics in non-recurrence group and recurrence group.

Variables	All (*n* = 180)	Non-recurrence group (*n* = 130)	Recurrence group (*n* = 50)	t/Z/χ^2^	*p*
Age	60.0(51.0, 67.0)	60.0(51.0, 67.0)	58.0(50.0, 66.0)	0.430	0.668
Male, n(%)	122(67.8)	87(66.9)	35(70.0)	0.157	0.692
BMI (IQR)	24.5(22.6, 27.2)	24.1(22.4, 27.6)	24.7(22.7, 27.0)	0.533	0.595
History of TIA or AIS, n(%)	48(26.7)	35(26.9)	13(26.0)	0.016	0.900
Other heart disease, n(%)	14(7.8)	8(6.1)	6(12.0)	1.721	0.190
History of myocardial infarction, n(%)	11(6.1)	7(5.4)	4(8.0)	0.431	0.512
History of diabetes, n(%)	37(20.6)	25(19.2)	12(24.0)	0.503	0.478
History of hypertension, n(%)	94(52.2)	71(54.6)	23(46.0)	1.074	0.300
Drink history, n(%)	48(26.7)	31(23.8)	17(34.0)	1.904	0.168
Smoke history, n(%)	70(38.9)	45(34.6)	25(50.0)	3.596	0.058
Posterior circulation ischemic, n(%)	85(47.2)	58(44.6)	27(54.0)	1.276	0.259
NIHSS (IQR)	1.0(0.0, 4.0)	1.0(0.0, 4.0)	2.0(0.0, 5.0)	1.011	0.288
TC (IQR)	3.8(3.2, 4.6)	3.6(3.1, 4.6)	4.0(3.4, 4.6)	1.576	0.115
TG (IQR)	1.2(0.9, 1.6)	1.2(0.9, 1.6)	1.3(0.9, 2.2)	1.183	0.237
LDL (IQR)	2.2(1.8, 2.9)	2.2(1.7, 2.9)	2.4(2.0, 2.8)	1.332	0.183
HDL (IQR)	1.0(0.8, 1.2)	1.0(0.8, 1.2)	1.0(0.8, 1.2)	0.500	0.618
ApoA1 (IQR)	1.1(1.0, 1.3)	1.1(1.0, 1.3)	1.1(1.0, 1.3)	0.760	0.448
ApoB (IQR)	0.8(0.7, 1.0)	0.8(0.7, 1.0)	0.9(0.7, 1.0)	1.624	0.105
FIB (IQR)	288.0(256.0, 334.0)	283.0(254.0, 331.0)	295.0(257.0, 345.0)	1.151	0.250
Ddimer (IQR)	0.6(0.5, 0.8)	0.6(0.5, 0.8)	0.7(0.5, 1.0)	0.704	0.477
HCY (IQR)	15.7(12.1, 20.3)	13.5(11.4, 18.2)	20.2(18.0, 22.8)	6.558	<0.001
GLU (IQR)	5.3(4.7, 6.3)	5.2(4.7, 6.3)	5.3(4.8, 6.2)	0.655	0.514
Stenosis (IQR)	0.5(0.4, 0.7)	0.4(0.4, 0.5)	0.7(0.6, 0.8)	6.825	<0.001
NWI (IQR)	0.8(0.6, 0.9)	0.7(0.6, 0.9)	0.9(0.8, 0.9)	4.350	<0.001
Positive remodeling, n(%)	60(33.3)	37(28.5)	23(46.0)	4.998	0.025
IPH, n(%)	46(25.6)	15(11.5)	31(62.0)	48.333	<0.001
Degree of enhancement, n(%)				30.322	<0.001
0	69(38.3)	64(49.2)	5(10.0)		
1	69(38.3)	47(36.2)	22(44.0)		
2	42(23.4)	19(14.6)	23(46.0)		

BMI, Body Mass Index; NIHSS, National Institute of Health Stroke Scale; TC, Total Cholesterol; TG, Triglyceride; LDL, Low-Density Lipoprotein; HDL, High-Density Lipoprotein; HCY, Homocysteine; GLU, Glucose; NWI, Normal Wall Index I; IPH, Intraplaque Hemorrhage.

### Feature selection for machine learning models

This study analyzed 27 variables using the LASSO regression of the training set data. To select the optimization parameter (λ) for the Lasso model, 10-fold cross-validation was performed based on the criterion of minimizing the standard deviation. The optimal λ value for LASSO, indicated by the vertical dashed line in Supplementary Figure S1, was found to be *λ* = 0.046. This *λ-*value corresponded to 7 features in the model, specifically the “history of hypertension” in baseline data, “homocysteine value” in laboratory tests, and “plaque features” obtained from high-resolution vessel wall magnetic resonance imaging, which included the “NWI value,” “stenosis rate,” “intraplaque hemorrhage,” “positive remodeling,” and “enhancement grade.”

### Machine learning model

**Figure 2:**
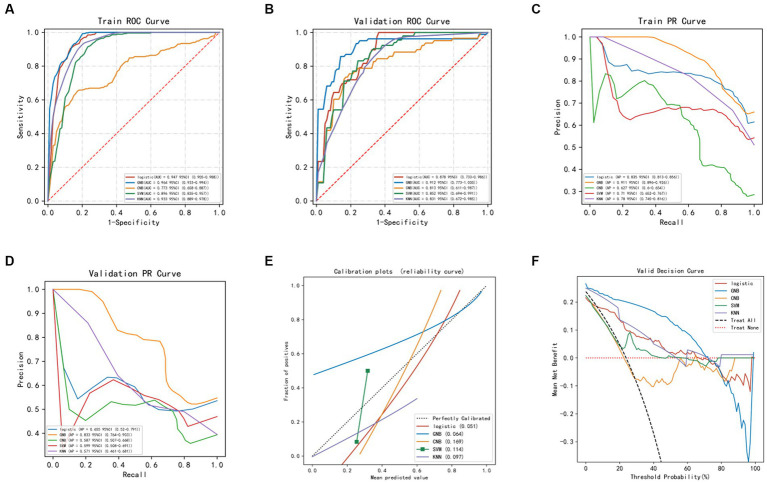
Evaluation of machine learning models. **(A)** ROC curve of machine models in the training set. **(B)** ROC curve of machine models in the validation set. **(C)** PR curve of machine models in the training set. **(D)** PR curve of machine models in the validation set. **(E)** Calibration curve of machine learning models in the validation set: The x-axis represents the average predicted probability, while the y-axis represents the actual probability of the event. The dashed diagonal line serves as the reference line, while the solid lines represent the fitting lines of different models. The closer the fitting line is to the reference line, the smaller the value inside the parentheses and the more accurate the model’s predictions. **(F)** DCA (Decision Curve Analysis) of machine learning models in the validation set: The black dashed line represents the hypothesis that all patients will experience stroke recurrence, while the red dashed line represents the hypothesis that no patients will experience stroke recurrence. The remaining solid lines represent different models.

The average accuracy values of the five machine learning models in the training set exceeded 0.64 (Table 2 and Supplementary Table S1), indicating their strong predictive ability. The GNB algorithm demonstrated promising performance in both the training set (AUC: 0.964, 95% CI: 0.933–0.994) and the test set (AUC: 0.912, 95% CI: 0.773–1.000), as depicted in Figure 2 and presented in Table 2. Our dataset showed an imbalance with a 5:13 ratio between the recurrent and non-recurrent stroke groups. As a result, we evaluated the precision-recall (PR) curve, which showed that the ROC curve is not an effective measure for assessing model efficacy when dealing with imbalanced data. The PR curve (Figures 2C,D) also demonstrated commendable performance for the GNB algorithm in both the training set (AUPRC: 0.896, 95% CI: 0.896–0.926) and the validation set (AUC: 0.833, 95% CI: 0.764–0.903). Subsequently, calibration curves and decision curve analysis (DCA) were employed to evaluate the predictive model (Figures 2E,F). The calibration plot of the validation set indicated minimal deviation between the predicted probabilities of recurrent stroke risk and the actual occurrence of events in the GNB model. DCA analysis revealed that the GNB model outperformed the other four models regarding clinical net benefit.

**Table 2:** Summarize the specific performance of the five machine learning algorithm models in the validation set.

Model	AUC	Accuracy	Sensitivity	Specificity	F1 score	Kappa
Logistic	0.878(0.733–0.986)	0.788(0.742–0.835)	0.975(0.926–1.000)	0.794(0.727–0.861)	0.598(0.414–0.782)	0.418(0.244–0.593)
GNB	0.912(0.773–1.000)	0.854(0.811–0.897)	0.923(0.870–0.977)	0.899(0.841–0.957)	0.763(0.685–0.841)	0.670(0.571–0.769)
CNB	0.813(0.611–0.987)	0.815(0.777–0.854)	0.793(0.678–0.909)	0.852(0.792–0.913)	0.653(0.585–0.722)	0.509(0.422–0.595)
SVM	0.852(0.694–0.991)	0.742(0.682–0.803)	0.935(0.882–0.988)	0.761(0.679–0.842)	0.617(0.529–0.704)	0.412(0.318–0.505)
KNN	0.831(0.672–0.985)	0.754(0.716–0.791)	0.955(0.919–0.991)	0.632(0.571–0.694)	0.704(0.575–0.832)	0.320(0.236–0.403)

### The construction and evaluation of the optimal GNB model

**Figure 3:**
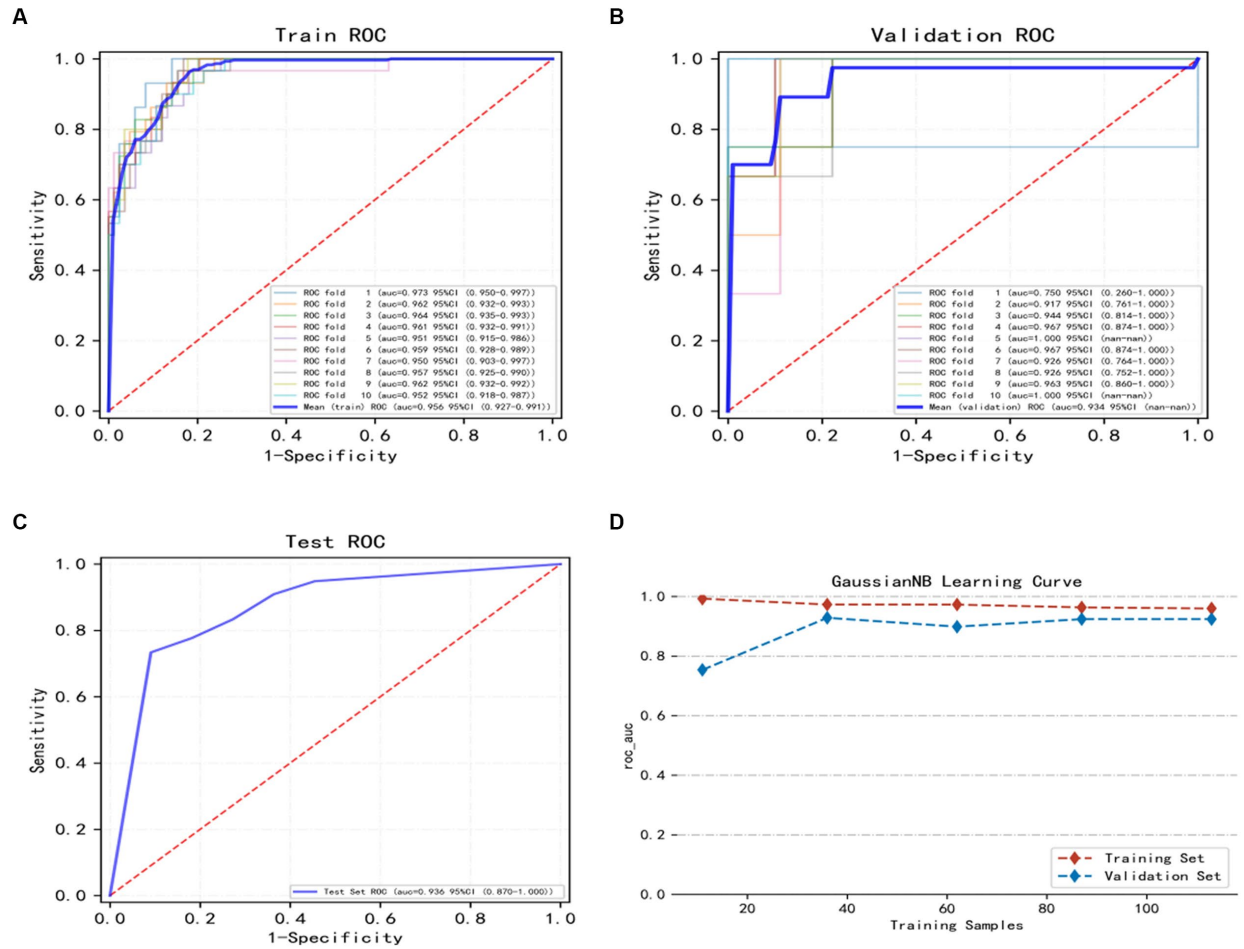
Illustrates the training, validation, and testing of the GNB model. **(A)** Training sets ROC and AUC and **(B)** validation sets ROC and AUC. Training and cross-validation of 10% of patients. Solid lines of different colors represent 10 different results. **(C)** Test set ROC and AUC. Test results for 30% of patients. **(D)** Learning curve. The red dashed line represents the training set and the blue dashed line represents the validation set. The values are expressed in terms of average and 95% CI.

The training set of the GNB model underwent 10-fold cross-validation. The results showed that the average AUC of the training set was 0.959 (0.927–0.991), the average AUC of the validation set was 0.934, and the average AUC of the test set was 0.936 (0.870–1.000) (Figures 3A–C), indicating a good predictive performance of the model. Considering that the performance of the validation set, as measured by the AUC metric, did not exceed or the ratio was lower than 10% from that of the test set, we deem that the model to have been successfully fitted (Figure 3D). Therefore, the GNB model can be applied to modeling tasks in relation to this dataset. In conclusion, the predictive model based on the GNB model algorithm exhibited the best performance in terms of prediction.

### Model interpretation

**Figure 4:**
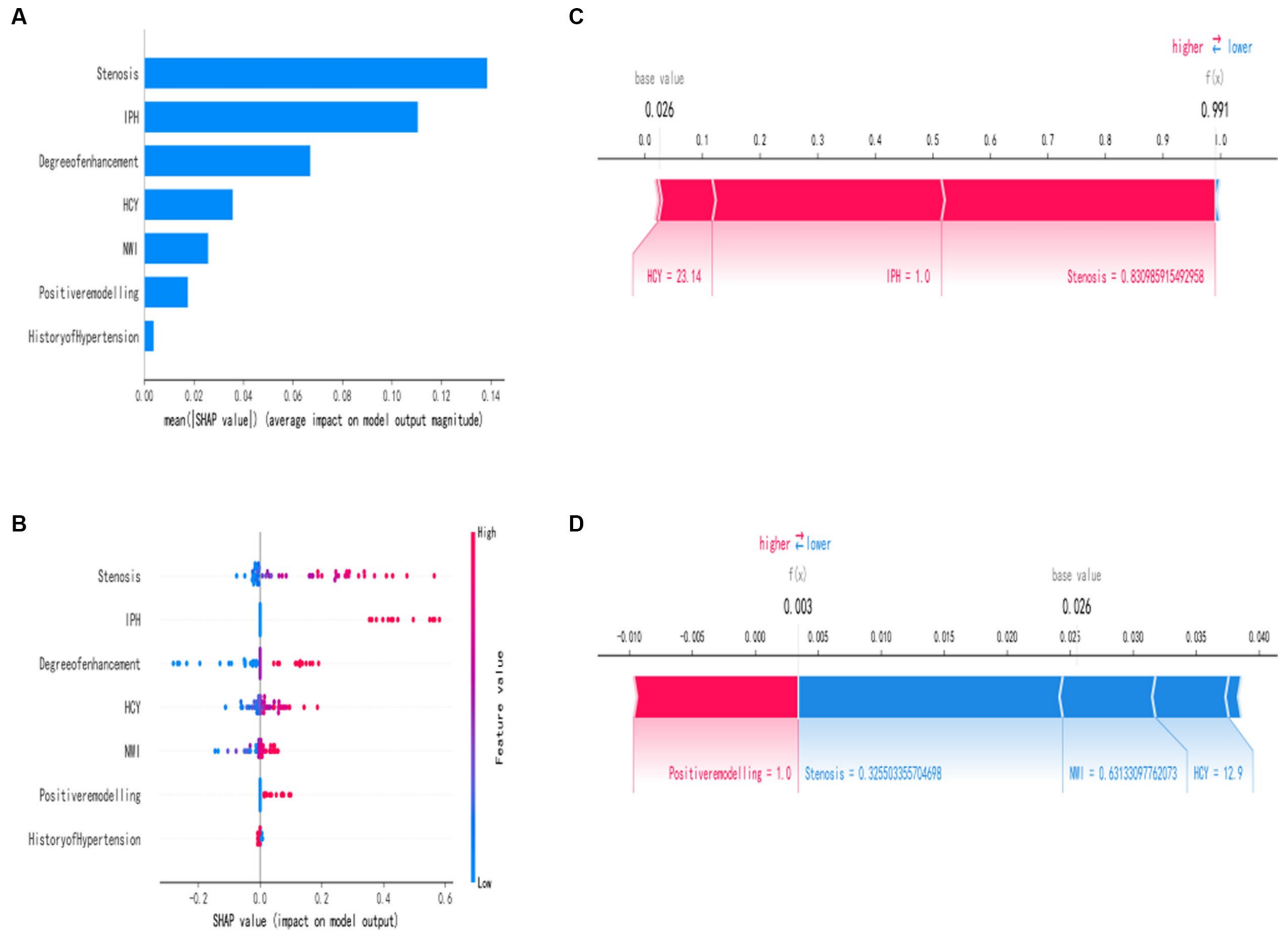
Interpretation of the best machine learning model (GNB) using SHAP. **(A)** Feature importance ranking represented by SHAP. The matrix plot describes the importance of each variable in the development of the final prediction model. **(B)** Feature attributes in SHAP. Each row represents a feature, and the x-axis represents the SHAP value. Red dots indicate higher feature values, while blue dots indicate lower feature values. **(C,D)** Individual risk explanations. Red features indicate an increased risk of death, while blue features indicate a decreased risk of death. The length of the arrows helps visualize the extent to which the predictions are influenced. The longer the arrow, the greater the effect.

In order to better explain how variables in the GNB model predict the occurrence of stroke recurrence, this study employed the SHAP method. Figure 4B illustrates the explanation of seven characteristics in the model. In each feature’s importance line, the attribution of all patients to the outcome is represented by dots of different colors, with red dots indicating high risk values and blue dots indicating low risk values. From the figure, it can be observed that an increase in plaque stenosis rate, plaque intraplaque hemorrhage, higher plaque enhancement level, elevated HCY levels, increased NWI values, the presence of positive plaque remodel, and a history of hypertension all contribute to an increased risk of stroke recurrence. Figure 4A presents a ranked bar graph of the contribution of the seven features to the model using mean absolute SHAP values. Finally, two typical cases are provided to demonstrate the interpretability of the model: Figure 4C shows a stroke recurrence patient, while Figure 4D shows a stroke no-recurrence patient.

## Discussion

With the advancements in modern medicine, the mortality and recurrence rate of acute ischemic stroke have significantly decreased compared to the past (Postma, 2019). However, stroke recurrence often leads to a poor prognosis, resulting in a reduced quality of life for patients and an increased economic burden. Research indicates that stroke recurrence is closely associated with a significant decrease in life expectancy (Peng et al., 2022). Hence, it is crucial to identify the risk factors for stroke recurrence to prevent, detect, and assess the prognosis.

This study developed a machine-learning model based on the GNB algorithm. The model incorporates simple baseline data on the history of hypertension, laboratory test results for homocysteine levels, and information about plaque from HR-VWI. The GNB model demonstrated superior performance to all other models in the training and validation sets. To enhance the interpretability of our machine learning model, we utilized SHAP summary plots to distinguish the importance of each feature.

Based on the SHAP plots, the model indicates that certain factors, namely the plaque stenosis rate, intraplaque hemorrhage, enhancement grade, and normalized wall index (NWI), are strong predictors of recurrent stroke in SICAS, consistent with previous studies. Ren et al. (2022) demonstrated the significant impact of plaque stenosis rate on stroke recurrence. From a hemodynamic perspective, plaque stenosis correlates negatively with perfusion in distant brain tissue and blood flow velocity. The stenosis of the vessel lumen, blood stasis, and increased pressure on the plaque significantly increase the risk of plaque rupture, thereby elevating the risk of stroke recurrence (Kim and Kim, 2014; Kwak et al., 2014). In our machine learning model, the plaque stenosis rate emerges as the most significant feature, reaffirming the findings of this study.

Additionally, intraplaque hemorrhage (IPH) and enhancement grade are identified as risk factors for stroke recurrence. Hosseini et al. (2013) and Kelly et al. (2020) demonstrate that IPH can be a new imaging biomarker for predicting stroke recurrence. These findings align with Ren et al. (2022), who report that patients with T1 high signal in responsible plaques are 2.878 times more likely to experience recurrence than non-T1 high signal patients. T1 high signal is often associated with plaque hemorrhage and lipid core. A meta-analysis comprising 13 studies (Schindler et al., 2020) indicates that IPH is the strongest predictor of stroke recurrence in symptomatic or asymptomatic carotid artery stenosis based on clinical features. IPH is associated with an increased risk of stroke at any degree of stenosis, even in patients with less than 50% stenosis. Furthermore, the enhancement grade of plaques, a common feature in HR-VWI, can help predict stroke recurrence. Typically, plaque enhancement is attributed to inflammation, neovascularization, and endothelial dysfunction, resulting in contrast agent leakage (Song et al., 2021). Kim et al. (2016) reveal that the 1-year stroke recurrence rate in plaques with enhancement is approximately five times higher than in non-enhancing plaques (30.3% vs. 6.8%). Song et al. (2021) report similar findings in a small study, where all 25 patients with acute ischemic stroke and carotid plaques with neovascularization experienced stroke recurrence, strongly supporting our viewpoint. The NWI value of plaques is also an important predictive factor. NWI is an index that measures plaque burden and significantly predicts plaque rupture and intraplaque hemorrhage. Ran et al. (2020) conclude that responsible plaques in the middle cerebral artery region increase the probability of stroke recurrence with a more significant plaque burden. By combining plaque burden with age and gender, their model predicts stroke recurrence with an AUC of 0.832, sensitivity of 72%, and specificity of 89%. Subsequently, Sun et al. (2021) obtained similar results across multiple vascular sites, demonstrating that a larger plaque burden is independently associated with stroke recurrence. Furthermore, plaque morphology is closely linked to recurrent stroke in SICAS (Zhao et al., 2017). Positive remodeling of plaques characterizes vulnerable plaques and can be explained pathologically as plaques containing larger lipid cores and more significant macrophage infiltration. Positively remodeled areas with larger external vessel wall areas often experience ruptured plaques, particularly in eccentric lipid plaques. Consequently, positively remodeled plaques bear a higher risk of stroke recurrence.

A history of hypertension is often a neglected element in routine medical history collection, even though it has been identified by Flach et al. (2020) and others as a risk factor for stroke recurrence, even after adjusting for relevant confounding factors. The measurement of homocysteine (Hcy) is a common laboratory test for stroke patients. Elevated Hcy levels can upregulate proinflammatory factors, leading to oxidative stress and ultimately causing vascular tissue remodeling. In a study conducted by Shi et al. (2018), it discovered that patients in the high Hcy group had a 1.76 times higher risk of recurrence than those in the low Hcy group. Subgroup analysis revealed a significant correlation between high Hcy levels and recurrence in patients with large artery atherosclerotic ischemia. Notably, the American Heart Association guidelines released in 2021 recommended using B vitamins as a preventive measure against stroke, reinforcing the reliability of our research.

In this study, the probability of stroke recurrence was 37.8%. Compared with other related studies, the higher recurrence rate suggests that it may be related to the fact that patients included in this study did not undergo relevant vascular interventional surgery. Although surgical treatment is not currently recommended as the primary option for SICAS patients in Chinese guidelines, recent research has shown that surgery combined with medication can effectively control the risk of stroke recurrence (Zheng et al., 2022; Zhong et al., 2022). Additionally, during the follow-up period, a certain proportion of patients in the study cohort did not follow the doctor’s instructions and regularly take medication, which may also contribute to the high recurrence rate of stroke. This further emphasizes the importance of dual antiplatelet therapy for the prevention and treatment of stroke recurrence (Zheng et al., 2022).

However, our study has several limitations. Firstly, the data for our machine learning algorithm model was only obtained from a single hospital, which may restrict its extensive implementation in other hospitals and clinical settings nationwide. Secondly, the study was constrained by a limited sample size due to regional restrictions. Lastly, as a retrospective study, inherent biases in the data are present. In order to address these limitations, we aim to conduct larger-scale, multicenter, and prospective studies in the future.

## Conclusion

In this study, we used a machine learning algorithm to develop five risk prediction models for predicting recurrent strokes in SICAS patients. We identified seven risk factors linked to recurrent strokes through clinical data, laboratory tests, and HR-VWI plaque characteristics screening. The GNB model showed the highest predictive accuracy, displaying high accuracy in both the training and validation sets and demonstrating outstanding clinical net benefits. This machine learning model aims to assist clinicians in personalized diagnosis and treatment, effectively preventing recurrent strokes in SICAS patients.

## Data availability statement

The raw data supporting the conclusions of this article will be made available by the authors, without undue reservation.

## Ethics statement

The studies involving humans were approved by the Ethics Committee of Xinxiang Medical University First Affiliated Hospital. The studies were conducted in accordance with the local legislation and institutional requirements. The participants provided their written informed consent to participate in this study. Written informed consent was obtained from the individual(s) for the publication of any potentially identifiable images or data included in this article.

## Author contributions

YG: Methodology, Writing – original draft. Z-aL: Data curation, Writing – review & editing. X-yZ: Software, Validation, Writing – review & editing. LH: Data curation, Writing – review & editing. PZ: Funding acquisition, Project administration, Writing – review & editing. S-jC: Writing – review & editing. J-yY: Methodology, Writing – review & editing. H-kC: Funding acquisition, Project administration, Supervision, Writing – review & editing.
