# Supplementary material for: An interpretable machine learning model for stroke recurrence in patients with symptomatic intracranial atherosclerotic arterial stenosis
Source: Front Neurosci. 2024 Jan 8;17:1323270. doi: 10.3389/fnins.2023.1323270 (PMC10800779; doi:10.3389/fnins.2023.1323270)
Supplement: Supplementary file 1 [file Data_Sheet_1.docx]

**Supplementary materials**.


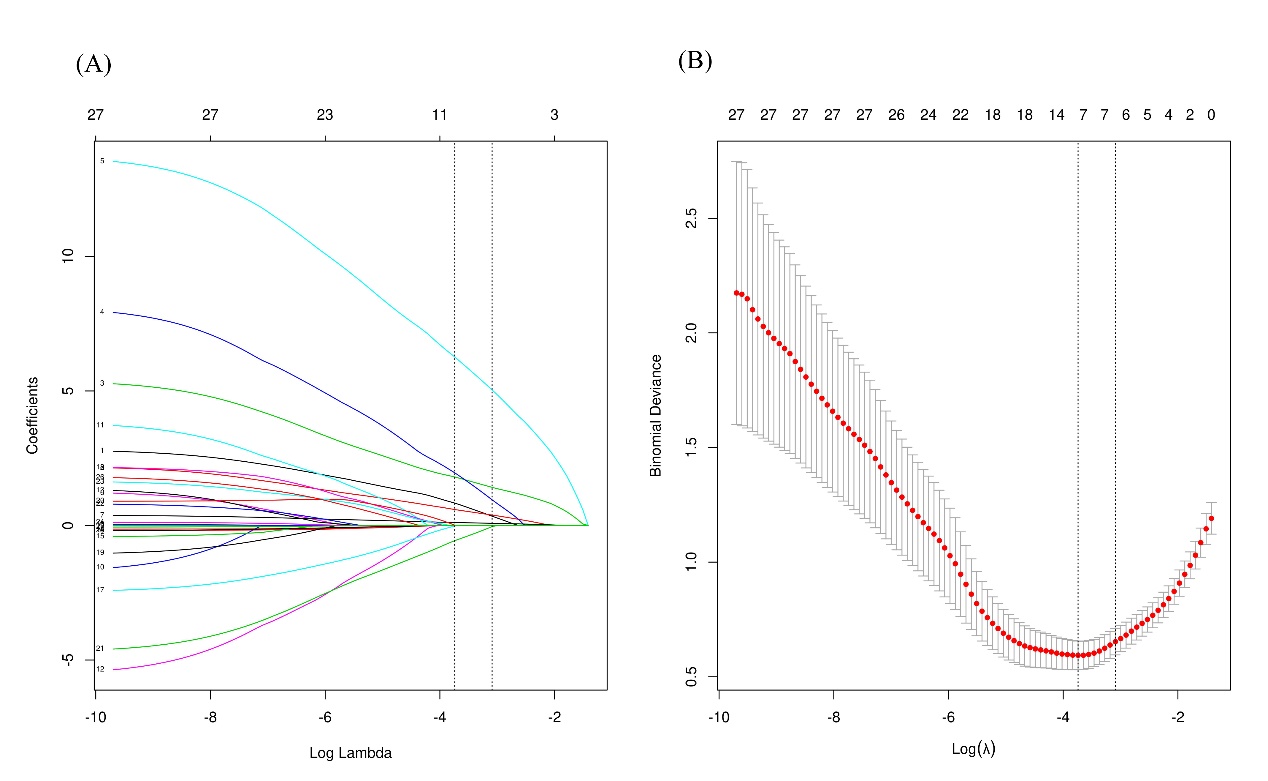


Supplement Figure 1 LASSO regression analysis was used to select characteristic factors. (A) displays the variations in 27 feature parameters with changing hyperparameter values (λ), representing the changes in different LASSO coefficient parameters. (B) illustrates the coefficient profiles of 27 texture features extracted from the log (λ) sequence in the LASSO model. Vertical dashed lines are drawn at the minimum mean square error (λ = 0.024) and the minimum standard error of distance (λ = 0.046).

Supplement Table2 Summarize the specific performance of the five machine learning algorithm models in the training set.

| Model | AUC | Accuracy | Sensitivity | Specificity | F1score | Kappa |
| --- | --- | --- | --- | --- | --- | --- |
| logistic | 0.947 (0.905-0.988） | 0.880(0.858-0.902) | 0.938(0.910-0.967) | 0.872(0.835-0.909) | 0.820(0.789-0.851) | 0.721(0.676-0.765) |
| GNB | 0.964 (0.933-0.994） | 0.861(0.846-0.876) | 0.993(0.980-1.007) | 0.828(0.806-0.851) | 0.795(0.773-0.817) | 0.685(0.655-0.716) |
| CNB | 0.773 (0.658-0.887） | 0.796(0.785-0.807) | 0.647(0.616-0.678) | 0.865(0.845-0.885) | 0.637(0.621-0.654) | 0.480(0.458-0.502) |
| SVM | 0.896 (0.835-0.957） | 0.839(0.802-0.876) | 0.931(0.891-0.970) | 0.818(0.760-0.876) | 0.771(0.731-0.812) | 0.644(0.578-0.709) |
| KNN | 0.933 (0.889-0.978） | 0.862(0.842-0.882) | 0.917(0.865-0.968) | 0.835(0.802-0.868) | 0.867(0.839-0.896) | 0.599(0.524-0.674) |
